# A Complex Interplay of Vitamin B1 and B6 Metabolism with Cognition, Brain Structure, and Functional Connectivity in Older Adults

**DOI:** 10.3389/fnins.2017.00596

**Published:** 2017-10-27

**Authors:** Kai Jannusch, Christiane Jockwitz, Hans-Jürgen Bidmon, Susanne Moebus, Katrin Amunts, Svenja Caspers

**Affiliations:** ^1^C. & O. Vogt Institute for Brain Research, Medical Faculty, Heinrich Heine University, Düsseldorf, Germany; ^2^Institute of Neuroscience and Medicine (INM-1), Research Centre Jülich, Jülich, Germany; ^3^Department of Psychiatry, Psychotherapy and Psychosomatics, Medical Faculty, RWTH Aachen University, Aachen, Germany; ^4^JARA–BRAIN, Jülich Aachen Research Alliance, Research Centre Jülich, Jülich, Germany; ^5^Institute of Medical Informatics, Biometry and Epidemiology, University of Duisburg-Essen, Essen, Germany

**Keywords:** gyrification index, FreeSurfer, aging, interindividual variability, resting state, cognitive performance, B vitamins

## Abstract

Aging is associated with brain atrophy, functional brain network reorganization and decline of cognitive performance, albeit characterized by high interindividual variability. Among environmental influencing factors accounting for this variability, nutrition and particularly vitamin supply is thought to play an important role. While evidence exists that supplementation of vitamins B6 and B1 might be beneficial for cognition and brain structure, at least in deficient states and neurodegenerative diseases, little is known about this relation during healthy aging and in relation to reorganization of functional brain networks. We thus assessed the relation between blood levels of vitamins B1 and B6 and cognitive performance, cortical folding, and functional resting-state connectivity in a large sample of older adults (*N* > 600; age: 55–85 years), drawn from the population-based 1000BRAINS study. In addition to blood sampling, subjects underwent structural and functional resting-state neuroimaging as well as extensive neuropsychological testing in the domains of executive functions, (working) memory, attention, and language. Brain regions showing changes in the local gyrification index as calculated using FreeSurfer in relation to vitamin levels were used for subsequent seed-based resting-state functional connectivity analysis. For B6, a positive correlation with local cortical folding was found throughout the brain, while only slight changes in functional connectivity were observed. Contrarily, for B1, a negative correlation with cortical folding as well as problem solving and visuo-spatial working memory performance was found, which was accompanied by pronounced increases of interhemispheric and decreases of intrahemispheric functional connectivity. While the effects for B6 expand previous knowledge on beneficial effects of B6 supplementation on brain structure, they also showed that additional effects on cognition might not be recognizable in healthy older subjects with normal B6 blood levels. The cortical atrophy and pronounced functional reorganization associated with B1, contrarily, was more in line with the theory of a disturbed B1 metabolism in older adults, leading to B1 utilization deficits, and thus, an effective B1 deficiency in the brain, despite normal to high-normal blood levels.

## Introduction

Aging is associated with cognitive decline as well as brain atrophy and functional brain network reorganization, particularly in higher association cortices of frontal, parietal, and temporal lobes. These changes are marked by a high inter-individual variability especially in older adults (Hultsch et al., 2002; Kane and Engle, 2002; Tisserand et al., 2002; Buckner, 2003; Brass et al., 2005; Grieve et al., 2007; Habib et al., 2007; Salthouse, 2009; Dickie et al., 2013). Among environmental and life style factors accounting for this high inter-individual variability at older age, nutrition seems to play an important role. Malnutrition has a strong impact on mental health in older adults, particularly due to hypovitaminoses; an aspect which has been underestimated for a long time (Wells and Dumbrell, 2006). It has repeatedly been shown, for example, that deficiency of B vitamins, namely vitamin B6 (Cochary et al., 1990; Malouf and Grimley Evans, 2006; Selhub et al., 2010) or vitamin B1 (Kuroki et al., 1993) are associated with cognitive deficits and structural brain changes during aging (Sturman and Rivlin, 1975; Selhub et al., 2000, 2010; Tucker et al., 2005).

For both, healthy as well as patient groups, beneficial effects of vitamin supplementation have been demonstrated, e.g., for diseases such as mild cognitive impairment, Alzheimer's disease (AD) or chronic alcohol abuse. First of all, there have been reports on improved cognitive outcome: Supplementation of vitamin B6 could improve verbal fluency in demented older adults. Patients with excessive alcohol consumption benefited from B1 supplementation regarding their executive functions (e.g., verbal fluency), working memory, and visuospatial coordination when being abstinent. In healthy older adults, back-to-normal levels of B1 have been shown to be beneficial for mood, reaction time, recall, attention and calculation, including global cognitive screenings such as Mini-Mental State Examination (Benton et al., 1995; Miller et al., 2002; Oscar-Berman et al., 2004; Mulder et al., 2005; Nardone et al., 2013; Lu et al., 2015) as well as for general cognition in AD patients (Lu'o'ng and Nguyen, 2011). In addition to these beneficial effects of vitamin B1 or B6 supplementation on cognition, effects in the brain have been reported as well. Low vitamin B1 and vitamin B6 blood levels were found to be associated with seizures, neurotransmitter dysfunction, white matter lesions and selective atrophy (Sturman and Rivlin, 1975; Purves et al., 2001; Mulder et al., 2005; Selhub et al., 2010). A higher level of daily intake of vitamin B6 was found to be related to higher gray matter volume in anterior cingulate and medial parietal cortex, middle temporal and superior frontal gyrus in healthy older adults (Erickson et al., 2008). In vitamin B1 deficiency, cortical (prefrontal cortex and basal forebrain) as well as subcortical (thalamic nuclei and mammillary bodies) volume loss has been reported in chronic alcohol abusers and patients suffering from Wernicke-Korsakoff syndrome (Pfefferbaum et al., 1997; Harding et al., 2000; Ratti et al., 2002; Oscar-Berman et al., 2004; Dirksen et al., 2006; Sullivan and Pfefferbaum, 2009). Here, supplementation of B vitamins has also been shown to be beneficial in that annual rates of brain atrophy were lowered (Jernerén et al., 2015).

Furthermore, utilization of vitamins B1 and B6 within the brain seems to differ. For vitamin B6, studies only reported beneficial effects of higher vitamin B6 levels on brain structure and cognition. For vitamin B1, utilization problems have been reported in older adults due to an insufficiently working active transport system through the blood-brain-barrier. A tight link between vitamin B1 blood levels and efflux rates across the blood-brain-barrier has been proposed. This leads to lower levels of active vitamin B1 metabolites and dependent enzymes in brain tissue even if vitamin B1 blood levels were within the normal range (Martin et al., 2003). This has also been found in brain tissue from patients with neurodegenerative diseases, even though their vitamin B1 blood levels were within the normal range (Lockman et al., 2003). No such utilization deficits in the brain have been reported for vitamin B6.

Thus, it could be assumed that even in the older population with normal blood levels, cognition as well as brain structure and functional network architecture might be differentially affected by these vitamins: higher vitamin B6 blood levels would be accompanied by preserved brain structure and stability of cognitive performance. In contrast, considering the utilization deficiency for vitamin B1 in older subjects, it might be hypothesized that different effects on cognition or brain structure might be observed, such as no or inverse relations. Related to that, it could be further assumed that also the functional network architecture might be differentially associated with blood levels of vitamins B6 and B1. The current study examined these differential relations of vitamin B1 and B6 level with brain structure and functional connectivity in a large population-based cohort of older adults with vitamin B6 and B1 blood levels within the normal range.

## Materials and methods

### Participants

Participants were recruited from the 1000BRAINS study, an epidemiological population-based cohort study designed to study influences of interindividual variability during brain aging in older adults (Caspers et al., 2014). 1000BRAINS is based on the epidemiologic population-based Heinz Nixdorf Recall Study and Heinz Nixdorf MultiGeneration study focusing on risk factors for atherosclerosis, cardiovascular disease, cardiac infarction and death (Schmermund et al., 2002).

The initial cohort of the current study consisted of 794 subjects aged between 55 and 85 years with available data on vitamin B1 and vitamin B6 blood levels. From this initial sample, subjects were excluded from further analysis due to: (i) more than three missing values in neuropsychological testing (*n* = 18); (ii) MRI datasets being incomplete or with failure during preprocessing, e.g., due to excessive motion (*n* = 108); and (iii) outliers of vitamin B6 and vitamin B1 blood levels (±2 standard deviations; *n* = 46). The final sample of the cortical structure analysis consisted of 657 adults aged 55–85 years (mean age: 67 years, 300 females and 357 males). For the functional connectivity analysis, additional 52 subjects were excluded due to incomplete functional MRI (fMRI) datasets or failure during pre-processing. This resulted in a final sample of 605 adults (mean age: 67 years, 279 females, and 325 males).

All subjects gave written informed consent prior to enrolment in the 1000BRAINS study according to the study protocol which was approved by the Local Ethics Committee of the University of Essen (Germany).

### Blood parameters

Vitamin B6 blood levels were measured as pyridoxal-5-phosphate and vitamin B1 blood levels were measured as thiamine-pyrophosphate (measurement unit: μg/l).

### Neuropsychological testing

Cognitive performance was measured using 14 different neuropsychological variables assessing cognitive functions in the domains of executive functions, attention, episodic and working memory, and verbal abilities (Table 1; for detailed information see: Caspers et al., 2014; Jockwitz et al., 2017). For subjects with not more than three missing values (subject with more than three missing values were excluded from the analysis), missing values were replaced by the median of the corresponding age and gender group (age-groups: 55–64 years, 65–74 years, 75–85 years). Since the neuropsychological data was not normally distributed, we rank-transformed all neuropsychological variables. Afterwards, the rank-transformed scores were mean centered and scaled (Jockwitz et al., 2017). These procedures were carried out using SPSS 21 (IBM).

**Table 1:** Neuropsychological variables used for the relation between vitamin B6 and cognition. Test explanation and associated cognitive functions of each neuropsychological test.

**Variable**	**Variables to measure**	**Cognitive functions**
Alters-Konzentrations-Test (Gatterer, 2008)	Time in seconds the participant needs to cross target variables	Selective attention
Wortschatztest (Schmidt and Metzler, 1992)	Number of correct identified real words in rows with 4 additional pseudo words	Language (vocabulary)
Trail Making Test A (taken from CERAD-Plus; Morris et al., 1989)	Time of connecting rising digits	Attention (processing speed)
Trail Making Test B-A (taken from CERAD-Plus; Morris et al., 1989)	Time differences between connecting rising digits (part A) and connecting alternately rising digits and letters (B)	Executive functions (concept shifting)
5-Punkte-Test (Jülicher version; similar to: Regard et al., 1982)	Number of singular marked figures by connecting 5 points Within 3 min	Executive functions (figural fluency)
Leistungsprüfungssystem 50+ (Subtest 3) (Sturm et al., 1993)	Number of correctly identified irregularities in a row of geometric figures within 5 min	Executive functions (problem solving)
Verbaler Gedächtnistest (Lux et al., 2012)	Number of reproduced words at 5 cycles after reading out a list of 15 words	Episodic memory (free recall)
Block-Tapping-Test (Schelling, 1997)	Number of correctly reproduced tapping sequences (sum score forward and backward)	Working memory (visual - spatial working memory)
Visual pattern (Jülicher Version; vergleichbar mit: Della Sala et al., 1997)	Number of reproduced patterns with increasing complexity given in a raster with black and white squares	Working memory (visual working memory)
Regensburger Wortflüssigkeitstest (Aschenbrenner et al., 2000)	Number of named words including the initial letter “B” within 2 min	Language (phonemic verbal fluency)
Regensburger Wortflüssigkeitstest (Aschenbrenner et al., 2000)	Number of named words associated with the category “profession” within 2 min	Language (semantic verbal fluency)
Benton-Test (Benton et al., 2009)	Number of total errors during free recall of 20 previously presented figures	Episodic memory (figural memory)
Zahlennachsprechen (nach Nürnberger Alters-Inventar; Oswald and Fleischmann, 1997)	Number of correctly repeated numbers of a previously presented sequence of numbers (sum score forwards and backwards)	Working memory (verbal working memory)
Farb-Wort-Interferenztest (Jülich version; similar to: Stroop, 1935; Bäumler, 1985)	Time difference in seconds between naming the ink of the written color words (part 3) and reading the color words (part 2)	executive functions (susceptibility to interference)

### Image acquisition

Structural and functional imaging data were obtained on a 3T Tim-TRIO MR scanner (Siemens Medical Systems, Erlangen, Germany), as part of the complete neuroimaging protocol of 1000BRAINS (Caspers et al., 2014). Structural brain imaging data were obtained using a 3D T1-weighted MPRAGE sequence (176 slices, *TR* = 2.25 s, *TE* = 3.03 ms, *TI* = 900 ms, FoV = 256 × 256 mm^2^, flip angle = 9°, final voxel resolution: 1 × 1 × 1 mm). Functional resting-state data were acquired using an echo-planar imaging (EPI) sequence (36 axial slices of 3.1 mm thickness, with *TR* = 2.2 s, *TE* = 30 ms, flip angle = 90°, in plane resolution: 3.1 × 3.1 mm^2^).

### Image data preprocessing

#### Local gyrification index analysis

T1 data were segmented into gray matter, white matter and cerebrospinal fluid using SPM8 (Statistical Parametric Modeling: www.fil.ion.ucl.ac.uk/spm). The combined gray matter + white matter mask was used for skull stripping of the brains to get rid of non-brain tissue voxels. These skull-stripped images served as input for processing within the FreeSurfer 5.1.0 pipeline via the recon-all command for automatic surface reconstruction and local gyrification index calculation (local gyrification; http://surfer.nmr.mgh.harvard.edu/; Dale et al., 1999; Fischl et al., 1999; Fischl and Dale, 2000; Schaer et al., 2008). Briefly, pre-processing of the T1-weighted anatomical images included motion correction, registration to a neuroanatomical Talairach atlas and intensity correction. This was followed by a segmentation of the brain into gray matter, white matter and cerebrospinal fluid. Using a tessellation procedure, a white matter surface mesh model was created (boundary between white and gray matter). To receive the pial surface, the mesh of vertices is inflated until the white matter surface reaches the boundary between the pial surface and the cerebrospinal fluid (Dale et al., 1999; Fischl et al., 1999).

After surface reconstruction, the local gyrification index (LGI) was calculated as implemented in FreeSurfer (Schaer et al., 2008, 2012). The gyrification index was initially defined by Zilles et al. (1988) in two-dimensional post-mortem coronal sections as the ratio between a contour along the pial surface and an outer surface covering the gyri but not going into the sulci. Schaer et al. (2008) extended this definition to a three dimensional LGI based on T1-weighted MR images. For each vertex on the pial surface, the LGI is calculated as the ratio between a region of interest (ROI) on the pial surface and a corresponding region of the outer hull surface (with a defined radius of 25 mm around the center vertex; Schaer et al., 2008). Thus, higher LGI is associated with higher local gyrification, and vice versa, lower LGI with reduced local gyrification. While gyrification overall is early determined during development, changes in the measure of the LGI was repeatedly interpreted as signs of local brain atrophy (Magnotta et al., 1999; Sallet et al., 2003; Bonnici et al., 2007; Zhang et al., 2010; Jockwitz et al., 2017) and is used as such in the present analysis.

#### Seed based resting-state analysis

Preprocessing of functional images was performed using SPM8 (www.fil.ion.ucl.ac.uk/spm). The preprocessing consisted of: (i) discard of the first four images; (ii) head motion correction using affine registration; (iii) spatial normalization of the mean EPI image to the MNI single subject template (Holmes et al., 1998) by using the unified segmentation approach (Ashburner and Friston, 2005) (iv) applying the ensuing deformation field to the individual EPI volumes; and (v) smoothing (5-mm FWHM Gaussian kernel). Afterwards, variance caused by nuisance factors that could lead to spurious correlations in the following RSFC analysis was removed from each voxel's time series (Weissenbacher et al., 2009; Jakobs et al., 2012; zu Eulenburg et al., 2012). This included the six motion parameters derived from the image realignment (head motion correction) as well as their first derivatives. These parameters were included as first and second order terms, resulting in 24 motion regressors (Satterthwaite et al., 2013). Furthermore, linear tissue regression of the mean gray, and white matter signal as well as cerebrospinal fluid was performed. In addition to that, artificial and counfounding signals were further removed by a PCA denoising (Behzadi et al., 2007; Hoffstaedter et al., 2014; Soltysik et al., 2015; Bright et al., 2017; Varikuti et al., 2017). Data were band-pass filtered to retain only those frequencies which contain relevant neuronal signal, i.e., between 0.01 and 0.08 Hz (Cordes et al., 2001). For the purpose of the current study, we examined the influence of B1 and B6 on resting state functional connectivity of the seed regions with all other gray matter voxels, while correcting for age, gender, head motion (using DVARS) and gray matter volume.

The statistical analysis was carried out using SPM8. Each significant cluster revealed from the structural analysis (vitamin B1/B6 × vertex–wise cortical folding) served as seed volume of interest (VOI) for the resting-state analysis. Each VOI was defined as the peak vertex of each significant cluster, with a 5-mm-sphere around it. Characteristic time-series of each seed region (see above of definition of seed regions based on the significant clusters from the structural analysis) was calculated for each subject by computing the first eigenvariate of the preprocessed time-series. To ensure that only gray matter signal was extracted from the VOIs, we only extracted signal of those 50% of the seed's gray matter voxels (median split) that had the highest probabilities of representing gray matter (according to the SPM8 segmentation). Afterwards, linear (Pearson) correlation coefficients between the VOI's time series and time series of all other gray matter voxels were calculated and transformed into Fisher's Z-scores.

### Statistical analysis

#### B vitamin level impact on cognition

First, blood levels of vitamin B6 and vitamin B1 were related to 14 different cognitive functions in the domains of attention, executive functions, language and episodic and working memory using partial correlation analyses, with age and gender added as covariates of no interest (implemented in SPSS 21.0, IBM Corporation). Results were significant at *p* < 0.05, FDR-corrected for multiple comparisons using the false discovery rate (FDR; (Benjamini and Hochberg, 1995). In addition, we performed an extreme group comparison of subjects with low and high vitamin B6 and vitamin B1 levels (*p* < 0.05, FDR-corrected for multiple comparisons.

#### B vitamin level impact on cortical structure

The correlation between blood levels of vitamin B6 and vitamin B1 and vertex-wise local gyrification was examined using general linear models (GLM) and Monte Carlo simulations for generation of the null-hypothesis using Qdec (http://surfer.nmr.mgh.harvard.edu/). For both analyses, age and gender were included of covariates of no interest. Results were reported at two different significance levels, i.e., *p* < 0.01 and *p* < 0.05 (FDR-corrected for multiple comparisons) for sensitivity testing and demonstration of differential effects.

#### B vitamin level impact on functional connectivity

Linear (Pearson) correlation coefficients were calculated between time series extracted from each seed region (see above) and each gray matter voxel in the brain. The resulting correlation coefficients were then transformed into Fisher's Z-scores and a second-level analysis of variance (ANOVA) was used to test for consistency across subjects. For the purpose of the current study, we examined the influence of B1 and B6 on resting state functional connectivity of the seed regions with all other gray matter voxels, while correcting for age, gender, head motion (using DVARS) and whole-brain gray matter volume (computed during the “unified segmentation” as explained above) to correct for differences in gray matter atrophy between subjects. Results were corrected for multiple comparisons across voxels using family-wise error (FWE) at *p* < 0.05, additionally adjusted for the two separate analyses carried out for vitamin B1 and B6, giving a final of *p* < 0.025 (using the false discovery rate, FDR; Benjamini and Hochberg, 1995).

## Results

Blood levels of vitamin B6 (0–32.3μg/l; mean = 9.34 ± 5.74) and vitamin B1 (34–73 μg/l; mean = 53.07 ± 8.21) were within the normal to high normal range for all subjects included in the present study, without significant correlation with age (B6: *r* = 0.014, *p* = 0.73; B1: *r* = −0.060, *p* = 0.14).

### Cognitive function in relation to vitamin blood levels

**Figure 1:**
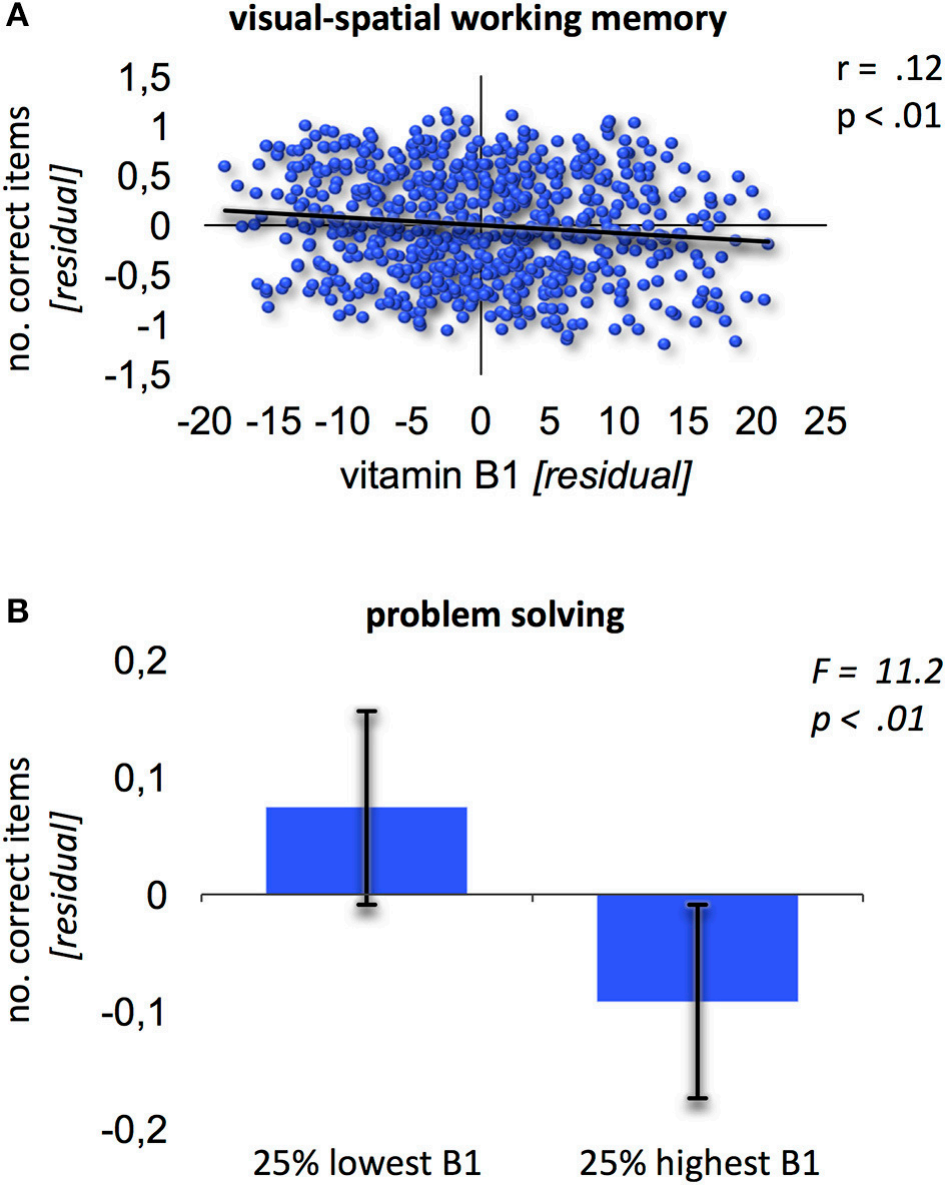
Relation of vitamin B1 and cognition. **(A)** Negative association between vitamin B1 and visual-spatial working memory (Block-Tapping-Test). Residuals derived from extracting age and gender from the scores. **(B)** Extreme group comparison between B1 and problem solving (Leistungsprüfungssystem-Test 50+, subtest 3). Error bars provide the standard deviation.

No significant effects were found when relating vitamin B6 blood levels to cognitive performance. Contrary, vitamin B1 was negatively related to performance in visual-spatial working memory (Block-Tapping-Test Schelling, 1997; Figure 1A; *r* = 0.12; *p* < 0.01, corrected for multiple comparisons). Thus, the higher vitamin B1 blood levels, the worse the performance in visual-spatial working memory. Additionally, the extreme group comparison showed better problem solving performance (Leistungsprüfungssystem 50+, subtest 3; Sturm et al., 1993) for subjects with 25% lowest high-normal vitamin B1 blood levels (Figure 1B; *F* = 11.2; *p* < 0.01).

### Relation between vitamin blood levels and local gyrification

**Figure 2:**
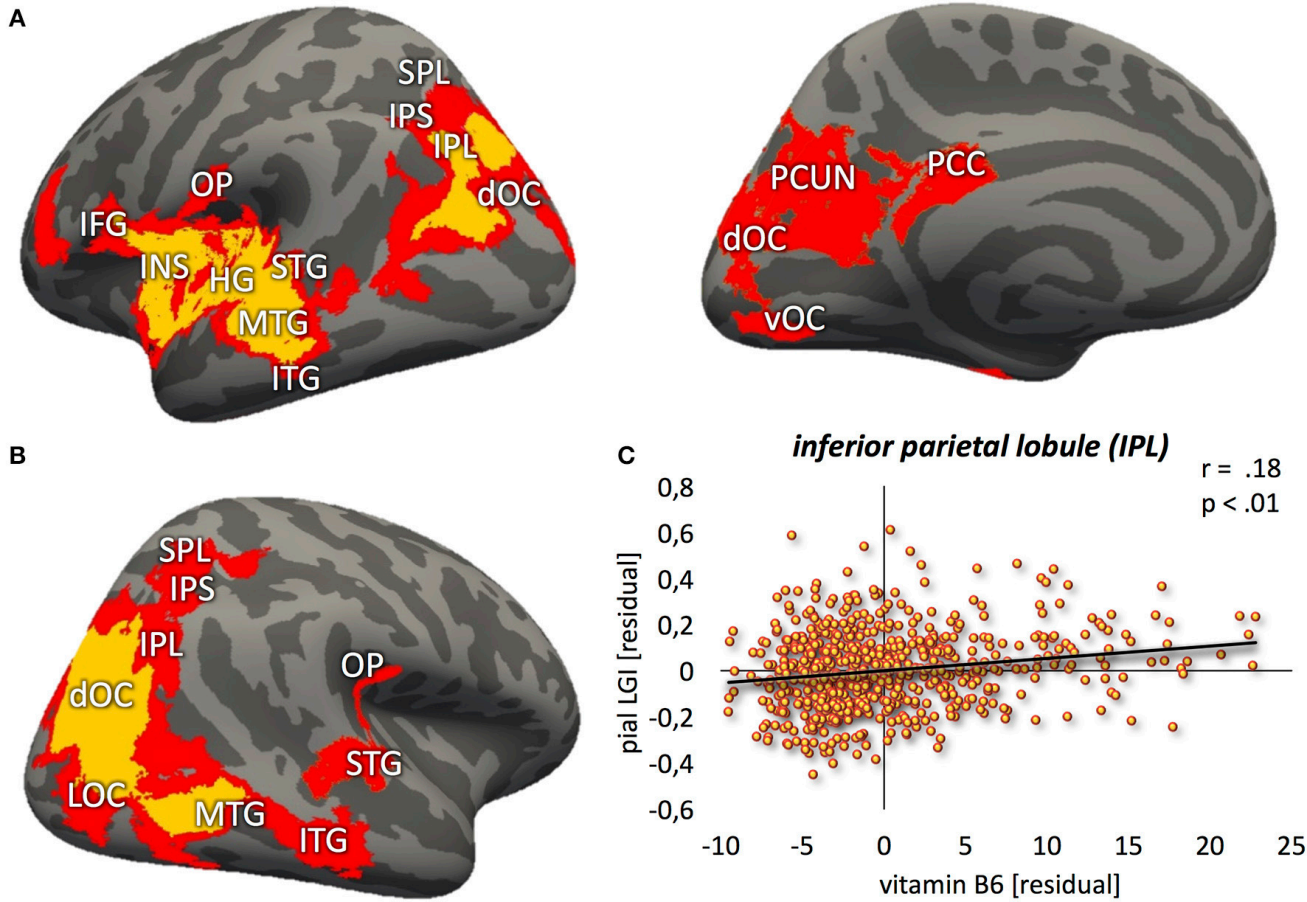
Positive relation between vitamin B6 and cortical folding (yellow: *p* < 0.01; red: *p* < 0.05) localized in the **(A)** left hemisphere (lateral-/medial view) **(B)** right hemisphere (lateral view). **(C)** Correlation between vitamin B6 blood levels and local gyrification index values, extracted from the most significant vertex cluster, i.e., left inferior parietal lobule (IPL). Residuals, corrected for age and gender. MFG, middle frontal gyrus; IFG, inferior frontal gyrus; INS, insula; HG, Heschl's gyrus; STG, superior temporal gyrus; MTG, middle temporal gyrus; ITG, inferior temporal gyrus; OP, parietal operculum; SPL, superior parietal lobe; IPS, intraparietal sulcus; dOC, dorsal occipital cortex; LOC, lateral occipital cortex; vOC, ventral occipital cortex; PCC, posterior cingulate cortex; PCUN, precuneus.

Vitamin B6 was positively correlated with higher LGI values (Figure 2; *p* < 0.01) within: (1) bilateral clusters comprised of inferior parietal lobule ranging into the visual cortex; (2) a large left perisylvian cluster including posterior and middle insular cortex, frontal and parietal operculum, inferior frontal gyrus and sulcus, and ventral somatomotor, auditory, posterior superior and middle temporal cortex as well as intraparietal sulcus ranging into the superior parietal cortex; and (3) a large right hemispheric cluster ranging into posterior parts of the inferior temporal lobe. These results were largely stable across different significance thresholds as revealed by the sensitivity analysis (Figure 2, red and orange overlays for *p* < 0.05 and *p* < 0.01, respectively; Table 2).

**Table 2:** Brain regions showing positive correlation between cortical folding and vitamin B6 blood levels.

	**Clusters at *p* < 0.01**		**Additional regions at *p* < 0.05**	
	**Macro-anatomy**	**Cytoarchitecture**	**Macro-anatomy**	**Cytoarchitecture**
**Vitamin B6**				
Left hemisphere			Middle frontal gyrus	
	inferior frontal gyrus	44 (Amunts et al., 1999, 2004)	inferior frontal gyrus	44 & 45 (Amunts et al., 1999, 2004)
			fusiform gyrus	
	insula	Ig1 & Ig2 & Id1 (Kurth et al., 2010)	insula	Ig1 & Ig2 & Id1 (Kurth et al., 2010)
	Heschl's gyrus	TE 1.0 & TE 1.1 & TE 1.2 (Morosan et al., 2001; Rademacher et al., 2001)	Heschl's gyrus	TE 1.0 & TE 1.1 & TE 1.2 (Morosan et al., 2001; Rademacher et al., 2001)
		TE 3 (Morosan et al., 2005)		TE 3 (Morosan et al., 2005)
			rolandic operculum	
	superior temporal gyrus			superior temporal gyrus
	middle temporal gyrus		middle temporal gyrus inferior temporal gyrus	
	parietal operculum	OP1 & OP2 & OP3 & OP4 (Eickhoff et al., 2006a,b)	parietal operculum	OP1 & OP2 & OP3 & OP4 (Eickhoff et al., 2006a,b)
			superior parietal lobe	7M (Scheperjans et al., 2008a,b)
	inferior parietal lobule	PGp & PGa (Caspers et al., 2006, 2008)	inferior parietal lobule	PGp & PGa & PFcm & PFop (Caspers et al., 2006, 2008)
			intraparietal sulcus	hIP3 ((Scheperjans et al., 2008a,b))
	dorsal occipital cortex		dorsal occipital cortex	hOc1 (V1) & hOc2 (V2) (Amunts et al., 2000) hOc3d (V3d) & hOc4d (V3a) (Kujovic et al., 2013)
	ventral occipital cortex		ventral occipital cortex	hOc3v (Vp) (Rottschy et al., 2007)
			posterior cingulate cortex	
			precuneus	
			cuneus	
			calcarine sulcus	
Right hemisphere	inferior parietal lobule	PGp (Caspers et al., 2006, 2008)	fusiform gyrus	FG2 (Caspers et al., 2013)
			gyrus postcentralis	2 (Grefkes et al., 2001)
			superior temporal gyrus	
			middle temporal gyrus	
			parietal operculum	OP1 (Eickhoff et al., 2006a,b)
			superior parietal lobe	7PC (Scheperjans et al., 2008a,b)
			inferior parietal lobule	PGp & PGa (Caspers et al., 2006, 2008)
			intraparietal sulcus	hIP1 (Choi et al., 2006), hIP3 (Scheperjans et al., 2008a,b)
	lateral occipital cortex	hOc4l1 (V4lp) & hOc4l2 (V4la) (Malikovic et al., 2016)	lateral occipital cortex	hOc4l1 (V4lp) & hOc4l2 (V4la) (Malikovic et al., 2016)
			dorsal occipital cortex	hOc3d (V3d) & hOc4d (V3a) (Kujovic et al., 2013)

**Figure 3:**
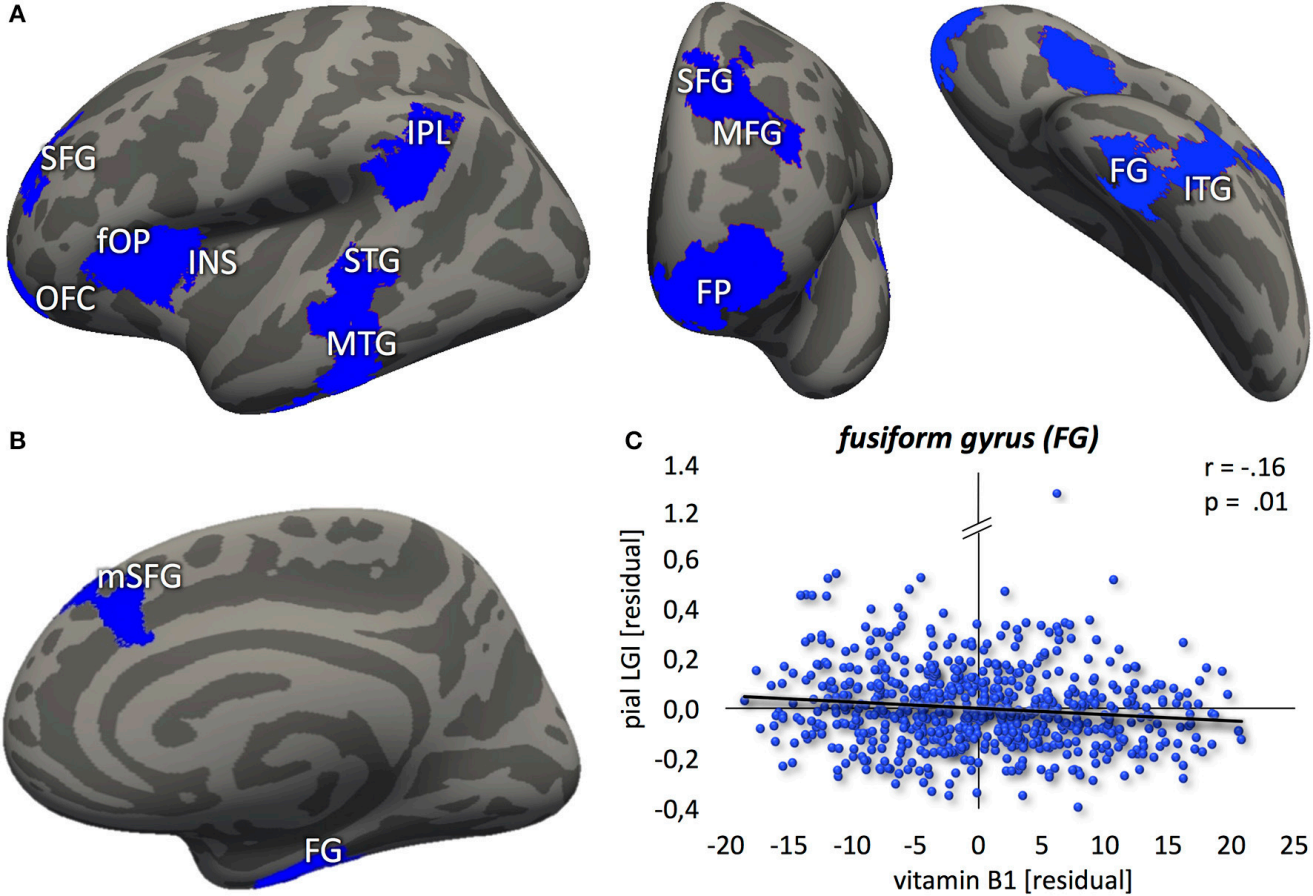
Negative association between vitamin B1 and cortical folding (blue: *p* < 0.05) localized in the **(A)** left hemisphere (lateral-/frontal-/caudal view) **(B)** right hemisphere (medial view). **(C)** Correlation between vitamin B6 blood levels and local gyrification index values, extracted from the most significant vertex cluster, i.e. the right fusiform gyrus (FG). Residuals, corrected for age and gender. FP, frontal pole; OFC, orbitofrontal cortex; SFG, superior frontal gyrus; mSFG, medial superior frontal gyrus; MFG, middle frontal gyrus; fOP, frontal operculum; INS, insula; IPL, inferior parietal lobule; STG, superior temporal gyrus; MTG, middle temporal gyrus; ITG, inferior temporal gyrus.

In contrast to that, no correlation between vitamin B1 and LGI was found at *p* < 0.01. Lowering the threshold to *p* < 0.05, however, revealed a negative association between blood levels of vitamin B1 and LGI in left frontal pole, anterior insula, dorsolateral prefrontal, middle part of the inferior parietal and posterior temporal cortex, and right dorsomedial prefrontal and anterior inferior temporal cortex (Figure 3). For a detailed description of significant brain regions at *p* < 0.05, see Table 3.

**Table 3:** Brain regions showing negative correlation between cortical folding and vitamin B1 blood levels.

	**Clusters at *p* < 0.01**		**Clusters at *p* < 0.05**	
	**Macro-anatomy**	**Cytoarchitecture**	**Macro-anatomy**	**Cytoarchitecture**
**Vitamin B1**				
Left hemisphere			frontal pole	Fp1 & Fp2 (Bludau et al., 2014)
			orbitofrontal cortex	Fo1 & Fo3 (Henssen et al., 2016)
			superior frontal gyrus	
			middle frontal gyrus	
			insular	
			Heschl's gyrus	TE3 (Morosan et al., 2005)
			superior temporal gyrus	
			middle temporal gyrus	
			inferior temporal gyrus	
			inferior parietal lobule	PF & PFm & PFcm & PGa (Caspers et al., 2006, 2008)
Right hemisphere			Medial superior frontal gyrus	FG3 (Lorenz et al., 2015)
			fusiform gyrus	
			entorhinal cortex	

### Relation between vitamin blood levels and functional connectivity

**Figure 4:**
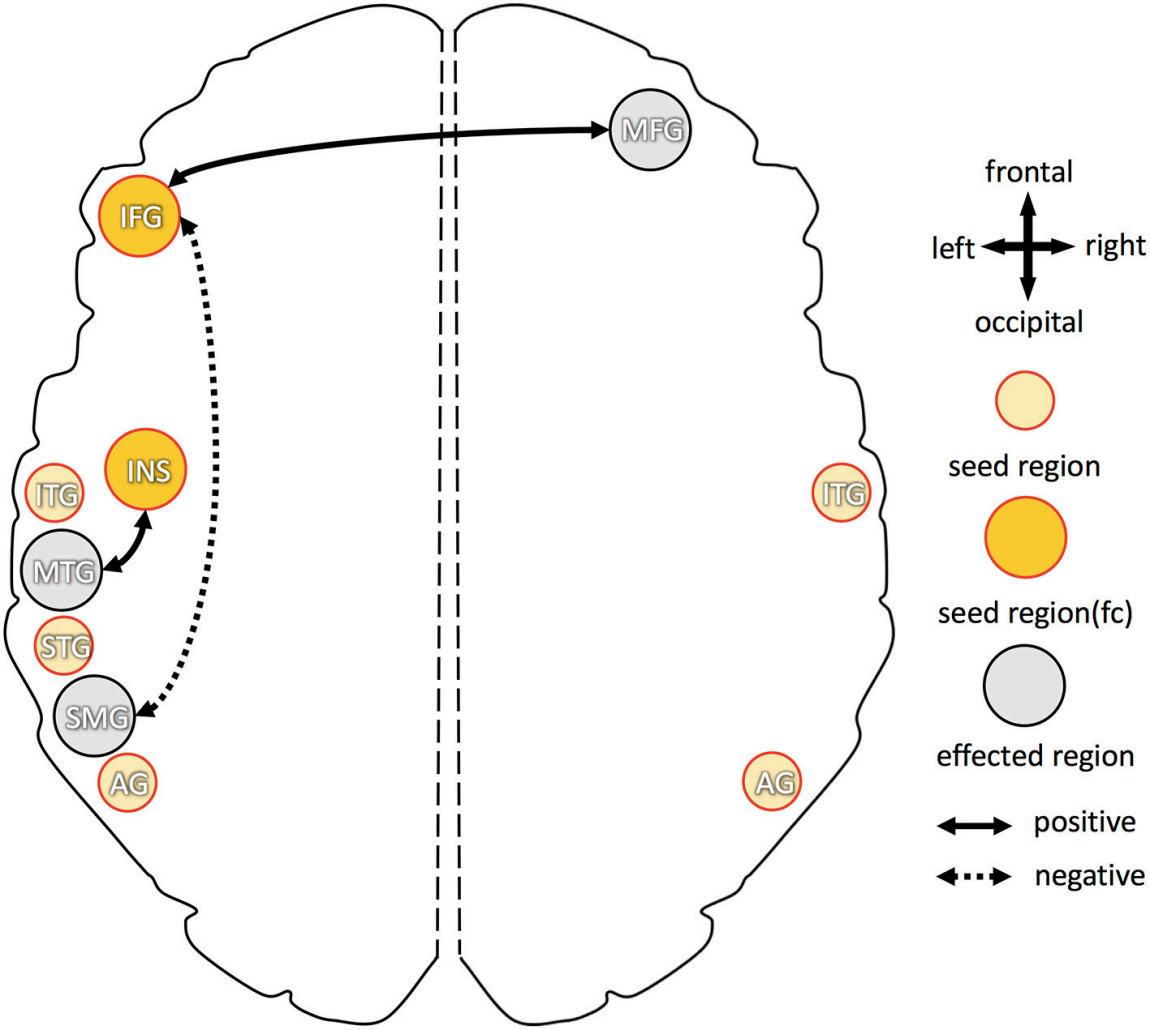
Overview of increased and decreased functional connectivity (FC) in association with vitamin B6 blood levels. Seed regions, derived from cortical folding analysis (peak vertices plus 5 mm sphere; see Figure 2), are depicted in yellow (intense yellow: if FC changes were observed; light yellow: if no FC changes were observed). Regions with of FC changes with one of the seed regions are marked in gray. Dotted arrows indicate decreased FC in association with higher vitamin B6 level, solid arrows show increased FC with higher B6. IFG, inferior frontal gyrus; MFG, middle frontal gyrus; INS, insula; STG, superior temporal gyrus; MTG, middle temporal gyrus; ITG, inferior temporal gyrus; SMG, supramarginal gyrus; AG, angular gyrus.

Higher B6 blood levels (Figure 4) resulted in stronger functional connectivity between: (1) left inferior frontal gyrus and right middle frontal gyrus; (2) left insula and left middle temporal gyrus. Additional decrease in functional connectivity was detected between left inferior frontal gyrus and left supramarginal gyrus in participants with higher B6.

**Figure 5:**
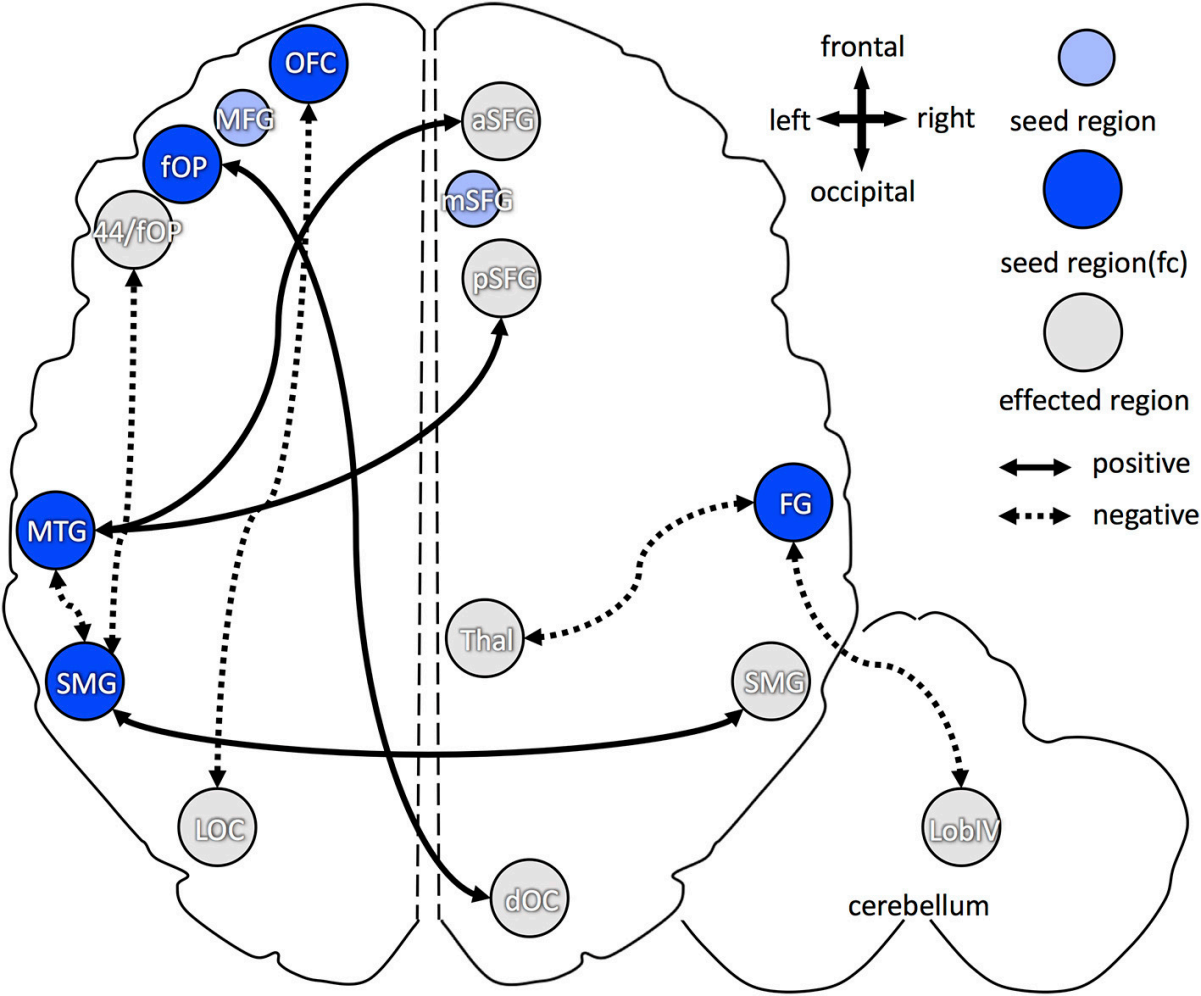
Overview of increased and decreased functional connectivity (FC) in association with vitamin B1 blood levels. Seed regions, derived from cortical folding analysis (peak vertices plus 5 mm sphere; see Figure 2), are depicted in blue (intense blue: if FC changes were observed; light blue: if no FC changes were observed). Regions with of FC changes with one of the seed regions are marked in gray. Dotted arrows indicate decreased FC in association with higher vitamin B1 level, solid arrows show increased FC with higher B1. OFC, orbitofrontal cortex; aSFG, anterior superior frontal gyrus; mSFG, medial superior frontal gyrus; pSFG, posterior superior frontal gyrus; fOP, frontal operculum; 44, Broca's region (area 44); MTG, middle temporal gyrus; FG, fusiform gyrus; SMG, supramarginal gyrus; dOC, dorsal occipital cortex; LOC, lateral occipital cortex; Thal, dorsomedian and pulvinar part of the thalamus; Lob IV, lobule IV of the cerebellum.

For higher B1 blood levels (Figure 5), positive association, i.e., increase in functional connectivity, was found between: (1) left frontal operculum and right dorsal occipital cortex; (2) left middle temporal gyrus and right anterior/posterior superior frontal gyrus; and (3) left and right supramarginal gyri. Decreased functional connectivity in participants with higher B1 was found between: (1) left orbitofrontal cortex and left lateral occipital cortex; (2) left middle temporal gyrus and left supramarginal gyrus; (3) left supramarginal gyrus and left frontal operculum (partially ranging into area 44); and (4) right fusiform gyrus and right thalamus (dorsomedial and pulvinar part) as well as right cerebellum (lobule IV).

## Discussion

We found pronouncedly differential effects of vitamins B1 and B6 on cognition, cortical atrophy and functional connectivity in a large sample of healthy older adults: while higher B6 levels were associated with better preservation of cortical structure throughout the brain, the effects on functional connectivity and cognition were rather small. For B1, contrarily, the higher the (already high-normal) blood levels, the worse the cognitive performance in problem solving and visual-spatial working memory and the more cortical atrophy in a distributed, mainly left-hemispheric network of prefrontal, inferior parietal and middle temporal areas. Additionally, a pronounced effect of high-normal B1 levels on functional connectivity was found, particularly on interhemispheric connections. This might indicate increased cross-talk between left-hemispheric brain regions showing B1 level related brain atrophy and largely non-affected brain regions in the contralateral hemisphere.

With regard to the following discussion, it needs to be stressed that vitamin B1 and B6 blood levels of the older subjects in the present study were within the normal to high-normal range. This is not a given as increasing age has previously been associated with decreases in vitamin B blood levels possibly due to metabolic and physiological changes, such as stomach acid production, atrophic gastritis, cirrhosis or reduced metabolites (Reinken and Zieglauer, 1978; Hallert et al., 1983; Henderson et al., 1986; Krasinski et al., 1986; Russell et al., 1986; Cochary et al., 1990; Ribaya-Mercado et al., 1990). Thus, any effects found in the present study should be interpreted from this perspective as previous studies on the effects of B vitamins mainly focused on subjects with pronounced vitamin deficiency.

### The opposing effects of vitamins B1 and B6 on cognition and brain structure

#### Vitamin B6–positive effects on brain structure

Lower levels of vitamin B6 in patients suffering from mild cognitive impairment (MCI) or Alzheimer's disease (AD) resulted in a worse course of disease (Miller et al., 2002; Mulder et al., 2005; Lu'o'ng and Nguyen, 2011). Supplementation of vitamin B6 revealed overall positive effects on cognitive function in these patient groups (Tucker et al., 2005; de Jager et al., 2012; Zhang et al., 2017), although null-effects in AD patients have also been reported (Aisen et al., 2008). Bryan et al. (2002) found better verbal fluency after supplementation of B6 in older adults with very low B6 blood levels. Studies investigating healthy, non-deficient adults showed contradicting results, though (for review, e.g., Malouf and Grimley Evans, 2006; Reay et al., 2013). While some authors did not report any effects of vitamin B6 on cognitive performance, others showed a positive correlation even in healthy older adults (Deijen et al., 1992; Kado et al., 2005; Balk et al., 2007; Harris et al., 2015; Krause and Roupas, 2015). Interestingly, Harris et al. (2015) reported a dissociation of vitamin supplementation effects in healthy older adults: while blood levels could be improved, no effect on cognitive performance was found. These previous studies thus hint at a ceiling effect of B6 supplementation for cognition: while cognition improved in neurodegeneratively diseased conditions or deficiency states, the amount of improvement slows down or even vanishes in healthy older subjects when normal to high-normal B6 blood levels are reached. Our present results add particularly to this latter aspect, in that we also found no effect on cognitive performance in relation to increasing B6 blood levels in our sample of healthy older adults with normal to high-normal B6 levels.

Regarding the effects of B6 levels on brain structure, a generally positive effect of vitamin B6 on neuronal development and growth was emphasized (Kirksey et al., 1990). Previous studies in patient populations yielded a similar picture as described for the B6 effects on cognition: supplementation of B6 led to better preserved gray matter in patients with MCI and Alzheimer's disease (Smith et al., 2010; Douaud et al., 2013). In contrast to the non-effects on cognition increases in B6 levels in healthy older adults were also associated with less gray matter atrophy (Erickson et al., 2008). With the body of literature on the association between B6 blood levels and brain structure being less extensive as compared to the evidence on the effects on cognition, our present study supports the previous notion on positive effects of B6 blood levels on preservation of brain tissue, showing that healthy older adults with normal to high-normal B6 blood levels have extended positive effects on cortical folding throughout the brain.

Taken together, our results amend previous knowledge on the effects of vitamin B6 on cognition and brain structure in healthy older adults: while preservation of brain surface structure, measured here via the gyrification index, were detectable throughout the brain even at normal to high-normal blood levels, cognition did not improve. This might be interpreted as vitamin B6 playing more a stabilizing or protective role: cognitive abilities might be stabilized at a constant level (but not additionally improved as seen in vitamin deficient subjects), while brain structure might be better preserved overall, potentially counteracting gray matter loss frequently occurring in older age. Future longitudinal studies as well as studies in independent samples are needed to further elucidate how vitamin B6 levels relate to brain structure and cognition in later decades of life.

#### Vitamin B1–negative association with both cognition and brain structure

In contrast to the non-effects of B6 on cognition and the positive association of B6 with cortical folding throughout the brain, we found a negative relation between vitamin B1 blood levels and cognitive performance in the domains of executive functions and working memory. We furthermore found no effect (at *p* < 0.01) or a negative association with cortical folding in a fronto-parieto-temporal brain network (at *p* < 0.05), respectively.

At first sight, this seems at odds considering results from previous studies. Nardone et al. (2013) showed decreases in spatial memory performance in adults with lower vitamin B1 blood levels, while Lu et al. (2015) found a weak positive association between B1 blood levels and general cognition in older subjects. Long-term vitamin depletion, including B1, e.g., due to alcohol abuse, led to impaired problem solving and (visual) working memory abilities (Butters et al., 1987). Thus, previous studies in patients reported a problem for low, but not for high B1 levels, the latter for the same functions as found to be affected in the present study on older people with high-normal B1 levels. Similar results were found for the association between B1 levels and brain structure. For example, a deficit in vitamin B1 blood levels seem to be associated with decreases in brain volume within e.g., the basal forebrain, frontal cortex, hippocampus and mammillary bodies in patients suffering from alcohol-dependency and Wernicke-Korsakow syndrome (Mair et al., 1979; Mayes et al., 1988; Pfefferbaum et al., 1997). Brain atrophy was furthermore described in patients suffering from neurodegenerative diseases, such as Alzheimer's- and Parkinson's disease (for a recent review see: Jhala and Hazell, 2011). In addition, vitamin B1 deficiency was associated with a downregulation of cholinergic transmission as well as cell loss of 25–45% in the basal forebrain (Arendt et al., 1995; Nardone et al., 2013), which seems to be related to successful performance in executive functions (for a recent review, see Wallace and Bertrand, 2013), decreases in cholinergic transmission might particularly affect the prefrontal cortex, as shown e.g., in alcohol-dependent patients (Pfefferbaum et al., 1997; Reuter-Lorenz et al., 2000; Ratti et al., 2002; Oscar-Berman et al., 2004; Dirksen et al., 2006; Nardone et al., 2013). Comparable relations were found between loss of acetylcholine and reduced cortical thickness within superior and inferior temporal gyri (Kilimann et al., 2016), regions which were also found to be affected in the present study. Generally, the regions showing reduced cortical folding in relation to higher vitamin B1 blood levels in the present study are part of a strongly interconnected fronto-parieto-temporal network (Caspers et al., 2011), which is smaller, but largely overlapping with regions typically found to be altered during aging and Alzheimer's disease as revealed by multimodal imaging (for review, see Sheline and Raichle, 2013).

Thus, the present results of an adverse effect of B1 on cognition and brain structure resembled the situation typically associated with a B1 deficient state, despite the fact that the older subjects of the present sample all had normal to high-normal B1 blood levels. Such a situation could be assumed if B1 is not adequately metabolized, leading to an effective B1 metabolite deficiency in the target tissue, as e.g., the brain. Such utilization problems of B1, i.e., normal B1 blood levels are associated with low B1 metabolite levels in the brain, have been particularly described in older subjects as well as in the course of neurodegenerative disease. Particularly in healthy and demented older subjects, lower levels of active vitamin B1 metabolites and dependent enzymes were already reported in brains of healthy and demented older adults (Gibson et al., 1988; Bettendorff et al., 1996). Vitamin B1 is actively transported through the blood-brain-barrier via an active influx-efflux transporter, leading to a tight control of B1 levels in brain tissue (Lockman et al., 2003). Thus, if B1 is constantly transported into the brain as supply is sufficient (i.e., B1 blood levels are normal or high-normal), but cannot be utilized adequately as described above, the active transport mechanism would ensure that B1 is transported back into the blood out of the brain tissue. On the other hand, this transporter might suffer from reduced sensitivity, which subsequently led to production deficits of vitamin B1 dependent enzymes and metabolites in the brain (Martin et al., 2003). Taken together, both, the higher efflux rate of vitamin B1 at the blood brain barrier, together with the loss of vitamin B1 dependent enzymes and metabolites would lead to the paradox of higher vitamin B1 in the blood accompanied by lower vitamin B1 in the brain, as particularly observed in older subjects. Relating this to the current results, it might be the case that although we measured higher vitamin B1 levels in the blood, the brain tissue might be in a state of effective vitamin B1 deficiency. This could account for the negative association between B1 blood levels and cognitive function and cortical folding, respectively. This effect was not very pronounced as it was only visible at a more lenient threshold, pointing to a very mild form of effective vitamin B1 deficiency in the present sample of healthy older adults. Our results thus provide a behavioral and structural correlate for the repeatedly reported B1 utilization deficiency in older subjects, which was so far mainly supported by biochemical post-mortem tissue analysis.

### Functional reorganization particularly associated with vitamin B1

Despite the comparably ample evidence regarding the effects of B vitamins on cognition and brain structure, there is almost no insight into their effects on the functional architecture of the brain or any reorganization thereof. In the present study, we mainly found increases of interhemispheric functional connectivity, while within the hemispheres, functional connectivity was reduced. This differential effect on intra- vs. interhemispheric connectivity was particularly evident for vitamin B1.

For B1, all the seed regions obtained from the structural analysis showed cortical atrophy, which was associated with higher B1, the same situation as found here for the increase in interhemispheric functional connectivity in our sample of healthy older adults. During aging, more prefrontal regions are found to be activated in older subjects, as a consequence of loss of function in posterior cortical regions (PASA; Grady et al., 1994; Davis et al., 2008). For those over recruited anterior regions, additional increases in functional connectivity were observed in Alzheimer's disease (Jones et al., 2011), hinting at an additional integration of prefrontal regions in a widened network. Another common theory of functional reorganization in aging argues that loss of function in brain regions of one hemisphere leads to additional recruitment of contralateral regions, making the whole functional network more bilateral in older as compared to younger subjects (HAROLD; Grady et al., 2000; Cabeza, 2002; Nielson et al., 2002). The latter provides evidence for increased interhemispheric functional connectivity as a potential compensatory mechanism, to overcome functional loss within the respective brain regions typically associated with a specific network within a hemisphere. Similar effects have been observed as reorganization of cognitive networks after stroke, e.g., within the somatomotor and dorsal attention network, which was associated with a regain in performance (e.g., Bannister et al., 2015; Veldsman et al., 2017); and in Parkinson patients with cognitive impairment as compared to controls, where internetwork connectivity was increased particularly between the hemispheres (Baggio et al., 2015). The latter study furthermore revealed decreased intranetwork connectivity, which was mainly found as reduced intrahemispheric functional connectivity. Thus, increased interhemispheric connectivity due to loss of function within a given network seems to reflect a common reorganizational principle both during normal aging as well as in age-related diseases. Such upregulation of interhemispheric and downregulation of intrahemispheric connectivity was described as being a highly efficient way of network function improvement and stabilization of performance (Cabeza, 2002).

The present results could be interpreted in a similar way. Based on the assumption of an effective vitamin B1 deficiency in the present sample of older adults (see first part of the Discussion), subjects with high-normal B1 levels seemed to face a comparable situation as observed during aging or disease: the network associated with B1 levels showed local atrophy. It was described that indeed both local atrophy could be associated with either increased or decreased functional connectivity (Kalpouzos et al., 2012). This is in line with the disruption of intrahemispheric connections and a potential increase of interhemispheric connectivity as observed in the present functional connectivity analysis. As this is similar to the findings reported in neurodegenerative disease, the idea that particularly the changes of functional connectivity found here in association with B1 might reflect compensatory strategies in a somewhat deficient state. Additionally, the patterns observed here reflect patterns observed in aging (including the more pronounced recruitment of prefrontal regions), making the phenomenon of accelerated aging as a consequence of this effective B1 deficiency another possible explanation. This needs to be further elucidated in future studies.

Remarkably, the changes in functional connectivity associated with B6 are rather small. A reason for this might be that vitamin B6 did not lead to cortical atrophy, but rather preserved cortex structure. Thus, there might be only limited need for functional reorganization. This also needs to be further evaluated in dedicated studies.

## Conclusion

The present study pointed to a complex interplay between vitamin B status and brain structure, function and cognition in healthy older adults. For B6, a measurable positive association with cortical folding without a detectable improvement in cognitive performance and only slight changes in functional connectivity were found, hinting at a still positive, but not additionally beneficial effect of vitamin B6 in healthy older adults with normal B6 blood levels. Contrarily, B1 was associated with cortical atrophy and decreased performance in problem solving and visuo-spatial working memory, accompanied by pronounced alterations of the functional connectivity architecture, being most in line with the repeatedly suggested theory of utilization deficiency of B1 within brain tissue in older subjects with high-normal B1 blood levels. Thus, our study yielded neuroimaging correlates for the assumed effect of B vitamins on the brain in healthy older subjects. With B vitamins being heavily involved in the production of different neurotransmitters (see Kennedy, 2016 for a recent review), it might be speculated that the present results might also provide a structural-functional counterpart for dysbalances in neurotransmitter systems, which might be an interesting line for further multimodal research approaches.

## Author contributions

KJ, CJ, and SC processed the data and conducted the different analyses. SM, KA, and SC conceived and designed the study. SM provided advise on data interpretation with regard to epidemiological relevance. HB critically advised and contributed to the interpretation and discussion of the data with regard to B vitamin metabolism. KA and SC supervised the whole study. KJ, CJ, HB, SM, KA, and SC wrote the paper.

### Conflict of interest statement

The authors declare that the research was conducted in the absence of any commercial or financial relationships that could be construed as a potential conflict of interest.
